# Expression of relative-protein of hypoxia-inducible factor-1α in vasculogenesis of mouse embryo

**DOI:** 10.1186/2241-5793-21-4

**Published:** 2014-05-13

**Authors:** Xueyi Dong, Baocun Sun, Xiulan Zhao, Zhiyong Liu, Qiang Gu, Danfang Zhang, Nan Zhao, Jinjing Wang, Jiadong Chi

**Affiliations:** Department of Pathology, Tianjin Medical University, Tianjin, 300070 China; Department of Pathology, Tianjin Cancer Hospital, Tianjin Medical University, Tianjin, 300060 China; Department of Pathology, Tianjin General Hospital, Tianjin Medical University, Tianjin, 300052 China

**Keywords:** Embryo vasculogenic mimicry, Hypoxia, Hypoxia-inducible factor-1α, Linearly patterned cell apoptosis

## Abstract

**Background:**

Physiological vasculogenesis in embryonic tissues share some important features with pathological neoangiogenesis in tumors. Linearly Patterned Programmed Cell Necrosis (LPPCN) and Vasculogenic Mimicry (VM) have been reported in tumors. The term VM refers to the aggressive tumor cells with CD31-negative phenotype to form Periodic Αcid Schiff (PAS)-positive network, that mimics the pattern of embryonic vasculogenic networks. LPPCN had been observed in our laboratory, and served as a spatial infrastructure for VM and endothelium-dependent vessel formation. Studies have been shown that hypoxia-inducible factor-1α (HIF-1α) can induce tumor cells to form vessel-like tubes and express genes associated with VM. Therefore, an analogous investigation has been carried out to determine if these patterns existed in mouse embryonic vasculogenesis.

**Results:**

In this essay, the results demonstrated that the number of Linearly Patterned Cell Αpoptosis (LPCA), embryo Vasculogenic Μimicry (embryo VM), endothelium-dependent vessels, and relative-protein of HIF-1α expression all showed time-dependent tendencies on E5.5-E9.5 (*p* < 0.05). The proteins CD133, VEGF, Twist, E-cadherin, and Vimentin showed local plexus distribution on E6.5-E7.5 (*p* < 0.05).

**Conclusions:**

LPCA and embryo VM existed in embryonic vasculogenesis. The relative protein of HIF-1α regulated the mouse embryonic vasculogenesis.

## Background

Adequate nutrient and substrate supply is essential for normal fetal intrauterine development. Vasculogenesis and angiogenesis are two consecutive processes during embryonic development. Vasculogenesis, the formation of the first blood vessels, is achieved by the differentiation of pluripotent mesenchymal cells into hemangiogenic stem cells. The subsequent step, angiogenesis, is characterized by the development of new vessels from already existing vessels [1]. The initial formation of blood vessels in embryonic tissues is similar to that of pathological neoangiogenesis in tumors. For example, they proliferate, migrate and invade through extracellular matrix and they have the ability to access the host vasculature and recruit a blood supply. However, physiologic neovascularization is tightly regulated, both temporally and spatially, and tumor angiogenesis is characterized by uncontrolled neovascularization [2, 3]. Many of the genes upregulated by aggressive tumor cells, which are involved in angiogenesis and vasculogenesis, have been shown to be involved in development, such as Vascular Endothelial Growth Factor (VEGF), Erythropoietin-Producing Hepatocellular Carcinoma-A2 (EPHA2), and so on. The cDNA microarray results showed a genetic reversion to a pluripotent embryonic-like genotype in the highly aggressive tumor cells [4–6].

LPPCN and VM have been reported in our laboratory that participated in the tumor blood supply in a time-dependent trend [7]. Studies have been shown that HIF-1α is switched under hypoxic conditions. HIF-1α induces angiogenesis by regulating a cohort of molecules, such as VEGF, Flt-1, and so on. Interestingly, HIF-1α may promote tumor cell reprogramming into endothelium cell by regulating Twist and VE-cadherin [8, 9]. However, the involvement of these phenomena in embryonic vasculogenesis have not yet been identified.

In 1999, the concept of VM was introduced to describe the unique ability of highly aggressive tumor cells to form PAS-positive and CD31-negative cells with a capillary-like structure and matrix-rich patterned network that mimics the embryonic vasculogenic network [5]. In tubular-type VM, non-endothelial cell-lined tubes resembling blood vessels are identified [10]. The term LPPCN refers to tumor cells darkly stained with H & E staining distributed in patterns of lines and networks. The distribution is similar to that seen in VM networks and endothelium-dependent vessels. LPPCN showed a form of “initiative death” that tumor cells were formed under hypoxic microenvironment. Currently, the vacuity formed by LPPCN was assumed to provide basis for VM and endothelium-dependent vessels [7]. Consequently, we used Kunming mice as a model to observe these phenomena involved in the embryo and analyze the relationship between them.

## Results

### Embryo VM and LPCA in the embryo

**Figure 1:**
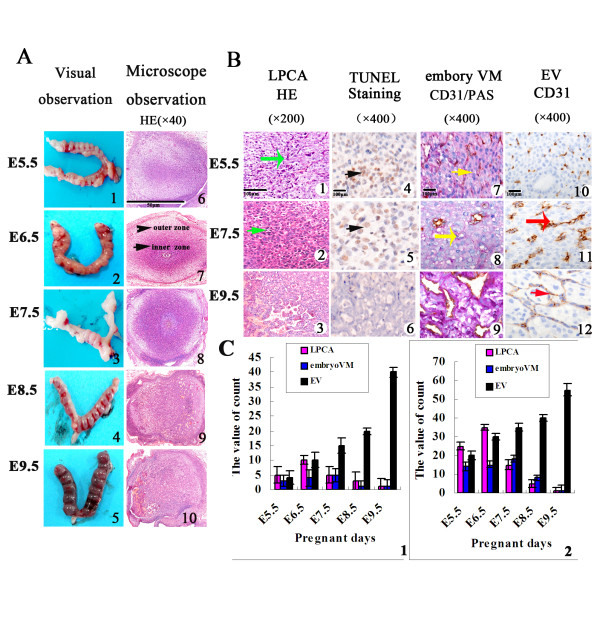
**Evidence of linearly patterned cell apoptosis and embryo vasculogenic mimicry in mouse embryonic vasculogenesis. (A)** Morphological observation of embryonic development by the naked eye (A1-A5) and under microscopy (A6-A10). The boundary between inner and outer zones is indicated by arrows. **(B)** Morphological observation of vessel pattern changed during E5.5-E9.5 in LPCA (H & E staining, ×200), embryo VM (CD31/PAS staining, ×400), and endothelium-dependent vessels (CD31 staining, ×400). Embryo cells (green arrow) displayed pyknosis, cytoplasm concentration, and chromatin condensation in LPCA areas. These darkly stained cells were distributed in lines and network patterns (B1-B3). The embryonic cells undergoing apoptosis displayed positive TUNEL staining (black arrows, ×400). They also had a distribution in lines and network patterns (B4-B6). The channels (yellow arrow) lined with embryo cells containing red blood cells was embryo VM. Embryo cells were negative for CD31 and positive for PAS staining (B7-B9). The endothelium-dependent vessels from E5.5 to E9.5 were shown by the red arrow (B10-B12). **(C)** The bar diagram represents the changed in LPCA, embryo VM, and endothelium-dependent vessels with time from E5.5 to E9.5 in the inner (C1) and outer zones (C2), respectively (*p* < 0.05).

Some embryo cells were darkly stained in the H & E stained sections on E5.5-E8.5. These darkly stained embryo cells arranged in 2-3 rows and there were approximately 20 cells in each row. Morphologically, these cells displayed pyknosis, cytoplasm concentration, and chromatin condensation. These cells were distributed in patterns of lines and networks. The distribution and morphological changes of these cells were similar to those in LPPCN in a tumor. Thus, we coined the term ‘LPCA’ to describe these darkly stained cells with special distribution patterns (Figure 1, B1-B3). The results also showed that the nuclei of LPCA were positive for TUNEL staining (Figure 1, B4-B6). Some vessel channels surrounded by embryo cells were negative for CD31 and positive for PAS staining. The channels were not composed of endothelial cells and red cells were found in it. Their structure was similar to that of VM in tumors. Hence, we coined the term ‘embryo VM’ to describe it (Figure 1, B7-B9). CD31 is a marker of endothelial cells, and the base membrane of endothelial cells is positive for PAS. Therefore, CD31 and PAS dual staining was used to distinguish embryo VM and endothelial-dependent vessels. We also observed endothelial-dependent vessels that were lined with both CD31-positive/PAS-positive cells (Figure 1, B10-B12). In summary, LPCA, embryo VM, and endothelium-dependent vessels existed in the embryonic development in the same period (Figure 1 and Table 1).

**Table 1:** **The value of linearly patterned cell apoptosis, embryo vasculogenic mimicry and endothelium-dependent vessels (**  **)**

	Linearly patterned cell apoptosis		Embryo vasculogenic mimicry		Endothelium-dependent vessels	
	Inner zone	Outer zone	Inner zone	Outer zone	Inner zone	Outer zone
E5.5	5 ± 0.58	25 ± 0.88	3 ± 0.58	14 ± 1.74	4 ± 0.58	20 ± 2.88
E6.5	10 ± 1.16	35 ± 2.89	4 ± 0.53	15 ± 1.15	10 ± 2.89	30 ± 5.19
E7.5	5 ± 1.05	15 ± 1.45	5 ± 0.88	18 ± 2.89	15 ± 1.45	35 ± 2.02
E8.5	4 ± 0.67	6 ± 0.75	1 ± 0.33	8 ± 1.73	20 ± 4.62	40 ± 4.62
E9.5	1 ± 0.55	1 ± 0.33	1 ± 0.47	1 ± 0.33	40 ± 4.74	55 ± 2.79
F-value	14.32	76.95	10.93	13.95	17.75	12.13
*p*	< 0.001	< 0.001	0.001	< 0.001	< 0.001	0.001

To determine the pattern of embryo blood supply at different stages of embryo growth, we measured the density of each type of vessel (MVD) daily from E5.5. The density of embryo VM was found to increase from E5.5 to E7.5, and then decreased from E8.5 to E9.5 in the inner zone and outer area, respectively. The density of LPCA peaked in E6.5, whereas that for embryo VM peaked on E7.5. MVD increased from E5.5 to E9.5. We observed elongated vessels from the periphery on E7.5, but most of them failed to mature. Vessels were formed in the networks in one terminal of the embryo on E9.5. Therefore, embryo VM was the major pattern of blood supply for embryonic growth at the early stages, and endothelium-dependent vessels dominated at the advanced stages of embryo growth. The change of LPCA, embryo VM, and endothelium-dependent vessels was time-dependent (*p* < 0.05) (Figure 1C and Table 1). LPCA may eventually form a channel-shaped, vessel-like empty space left by dead cells, as well as a supply space for embryo VM and endothelium-dependent vessels.

### Expression of CD133, HIF-1α, and VEGF at different stages of embryonic growth

**Figure 2:**
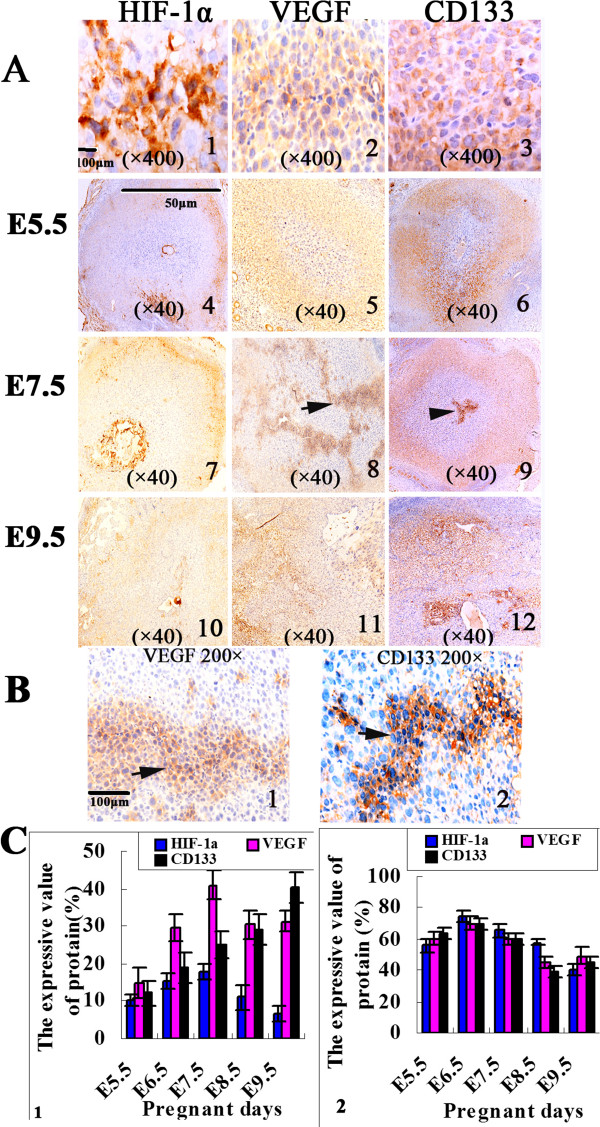
**Evidence of the expression of HIF-1α, VEGF, and CD133 proteins in mouse embryonic vasculogenesis. (A)** Morphological observations of HIF-1α, VEGF and CD133 protein expression changed in E5.5-E9.5 with IHC staining (A1-A3, ×400; A4-A12, ×40). HIF-1α positive expression in embryo cells was identified in the cytoplasm as well as in nucleolus (A1). VEGF positive expression in embryo cells was identified in the cytoplasm (A2), CD133 positive expression in embryo cells was identified in the cytoplasm and membrane (A3). A marked change between the inner and outer zones can be observed in the figure (A4-A12). **(B)** Morphological observations of CD133 and VEGF protein expression showed an irregular liner pattern in E6.5-E7.5 and the pattern was similar to endothelium-dependent vessels distribution with IHC staining (B1-B2, ×200). **(C)** The bar diagram represents the changed in the expression of the three proteins with time from E5.5 to E9.5 in the inner (C1) and outer zones (C2), respectively (*p* < 0.05).

By immunohistochemical staining, the expression of CD133 was in the cytoplasm and membrane of embryo cells, HIF-1α in the cytoplasm and nucleolus of embryo cells, as well as VEGF in the cytoplasm of embryo cells. The expression intensities of VEGF, HIF-1α and CD133 in the outer area were higher than those in the inner zone on E5.5-E6.5 (*p* < 0.05) (Figure 2A). On E7.5, the expression intensities of VEGF and CD133 showed local distribution significantly (Figure 2B). The highest VEGF expression was in the inner zone on E7.5 and in the outer zone on E6.5. The highest HIF-1α expression was in the inner zone on E7.5 and in the outer zone on E6.5 while the highest CD133 expression was in the outer zone on E6.5 and increased in the inner zone (*p* < 0.05). The level of HIF-1α expression decreased on E8.5-E9.5. VEGF and CD133 expression increased in E9.5 (*p* < 0.05) (Figure 2C and Table 2).

**Table 2:** **The expression of HIF-1α, VEGF and CD133 proteins in mouse embryonic vasculogenesis (**  **)**

	HIF-1α		VEGF		CD133	
	Inner zone	Outer zone	Inner zone	Outer zone	Inner zone	Outer zone
E5.5	10.0 ± 1.58	55.6 ± 4.04	15.0 ± 4.12	60.2 ± 3.96	12.0 ± 3.08	63.4 ± 4.22
E6.5	15.2 ± 1.92	74.8 ± 3.56	29.6 ± 3.65	70.0 ± 3.81	19.0 ± 4.18	69.6 ± 3.65
E7.5	17.8 ± 1.92	65.4 ± 3.85	41.0 ± 3.81	59.6 ± 3.21	25.0 ± 3.54	60.0 ± 3.54
E8.5	11.0 ± 3.39	57.6 ± 2.07	30.4 ± 3.64	45.0 ± 4.12	29.0 ± 4.18	39.0 ± 4.18
E9.5	6.60 ± 2.07	40.0 ± 3.81	31.2 ± 2.77	49.0 ± 5.43	40.4 ± 4.16	45.6 ± 3.78
F-value	18.96	66.36	32.88	28.28	54.02	38.54
*p*	< 0.001	< 0.001	< 0.001	< 0.001	< 0.001	< 0.001

### Expression of twist, E-cadherin, and Vimentin at the different stages of embryonic growth

**Figure 3:**
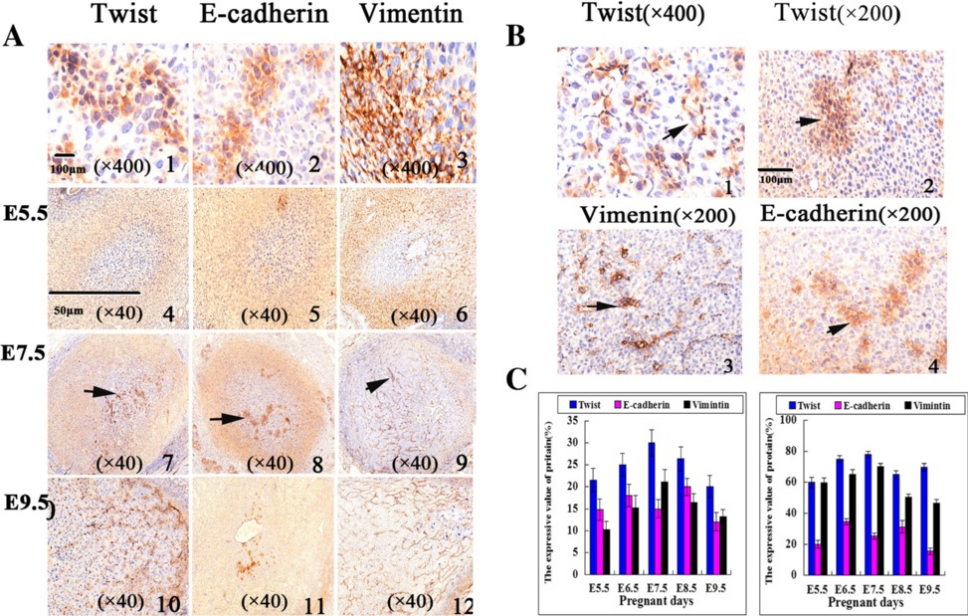
**Evidence of the expression of Twist, E-cadherin, and Vimentin proteins in mouse embryonic vasculogenesis. (A)** Morphological observation of the changes in the expression of Twist, E-cadherin, and Vimentin protein expression from E5.5 to E9.5 with IHC staining (A1-A3, ×400; A4-A12, ×40). Twist positive expression in embryo cells was identified in the cytoplasm as well as in nucleolus (A1). E-cadherin positive expression in embryo cells was identified in the cytoplasm and membrane (A2). Vimentin positive expression in embryo cells was identified in the interstitial tissue (A3). The marked change between the inner and outer zones can be observed in the figure (A6-A12). **(B)** Morphological observation of Twist protein expression in the adjacent area of vessels (B1, ×400). Morphological observation of Twist, E-cadherin, and Vimentin protein expression showed a patchy pattern during E6.5-E7.5 with IHC staining (B2-B4, ×200). **(C)** The bar diagram represents the changed in the expression of the three proteins with time from E5.5 to E9.5 in the inner (C1) and outer zones (C2), respectively (*p* < 0.05).

Immunohistochemical staining revealed the expression of E-cadherin in the cytoplasm and membrane of embryo cells, Twist in the cytoplasm and nucleolus of embryo cells, as well as Vimentin in embryonic interstitial tissues. The expression intensities of Twist, E-cadherin, and Vimentin in the outer area were higher than those in the inner zone on E5.5-E6.5 (*p* < 0.05) (Figure 3A). On E7.5, the expression intensities of Twist, E-cadherin, and Vimentin showed local distributions significantly (Figure 3B). Twist protein expression around the vessels was observed. The highest expression of Twist and Vimentin appeared in the inner and outer zones on E7.5 (*p <* 0.05). The highest E-cadherin expression was in the inner zone on E8.5 and in the outer zone on E6.5. The expression of Twist increased on E9.5, and E-cadherin and Vimentin expression decreased on E9.5 (*p* < 0.05) (Figure 3C and Table 3).

**Table 3:** **The expression of twist, E-cadherin and Vimentin proteins in mouse embryonic vasculogenesis (**  **)**

	Twist		E-cadherin		Vimentin	
	Inner zone	Outer zone	Inner zone	Outer zone	Inner zone	Outer zone
E5.5	21.6 ± 2.70	60.0 ± 3.39	14.8 ± 2.39	20.0 ± 2.54	10.2 ± 1.92	59.6 ± 3.04
E6.5	25.0 ± 2.55	74.8 ± 2.39	18.0 ± 2.55	34.6 ± 3.07	15.2 ± 2.86	65.2 ± 2.86
E7.5	30.0 ± 3.05	77.8 ± 1.92	15.0 ± 2.07	25.2 ± 1.92	21.2 ± 2.77	70.2 ± 1.92
E8.5	26.4 ± 2.70	65.0 ± 2.55	20.0 ± 1.92	31.2 ± 3.96	16.4 ± 2.07	50.4 ± 2.07
E9.5	20.0 ± 2.55	69.8 ± 2.39	12.0 ± 2.07	15.4 ± 2.07	13.2 ± 1.64	46.4 ± 2.41
F-value	21.91	39.19	60.31	134.67	15.61	89.05
*p*	< 0.001	< 0.001	< 0.001	< 0.001	< 0.001	< 0.001

## Discussion

Adequate nutrient and substrate supply is essential for normal fetal intra-uterine development. Embryonic angiogenesis involved a complex series of events during which endothelial cells differentiate, proliferate, migrate, and undergo maturation into an organized vascular network [11]. In mammals, vascular progenitors first appeared in the yolk sac, where extra-embryonic mesodermal precursors of both hematopoietic and endothelial lineages differentiate into solid clumps, which begin to form morphologically identifiable “blood islands” on E6-E6.5 [12]. The vasculogenesis of the embryo proper is initiated on E7-E7.5 [13]. In the rapid growth stage, the blood supply arrived the “blood islands” was not satisfied the oxygen and nutritional needs of embryonic development on E5.5-E7.5. Embryo proper cells have been suffered from anoxia on E5.5-E7.5.

Hypoxia is an elemental physiological stimulus that occurs in response to tissue growth during normal development [14]. Hypoxia occurs when oxygen supply is decreased and/or demand is relatively increased during normal development. A large number of genes involved in different steps and individual phenotypic processes of angiogenesis are known to be regulated by hypoxia. HIF-1α plays an important role in this process [15] and LPCA was formed under hypoxia conditions and regulated by a series of genes. We presumed that the moribund cells from LPCA may provide transient spatial infrastructure for embryo VM as well as endothelium-dependent vessel in developmental processes. At the same time, embryo cells around the LPCA remold to form embryo VM. Similar results have been observed in tumor by our earlier experiment [7, 16].

The results showed that embryo VM was the main pattern of blood supply for the embryo on E5.5-E7.5. As the embryo expanded, endothelial cells differentiated and proliferated. Consequently, endothelium-dependent vessels replaced embryo VM as the major pattern of blood supply on E8.5-E9.5. Presumably, this observation was based on the fact that LPCA, embryo VM and endothelium-dependent vessels existed in embryo by time-dependent manner.

The immunohistochemical data also revealed that regions of LPCA were positive for HIF-1α and negative for VEGF and CD133 on E5.5-E7.5. HIF-1α-VEGF axis regulating angiogenesis program plays a critical role in ischemic tissue and tumor [17, 18]. HIF-1a is a transcription factor selectively stabilized and activated under hypoxic conditions, and coordinates the adaptive response of tissues to hypoxia [19]. In our study, LPCA cells were intensively positive for HIF-1α in the development of embryo. The highest of LPCA was consistent with the highest expression of HIF-1α on E6.5. Our study suggested hypoxia relieved when embryo VM was formed after E7.5. At the same time, the embryonic cells around the LPCA were positive for VEGF and CD133. VEGF signaling is the best-known pathway that regulates the formation and morphogenesis of blood and lymphatic vessels during development. During development, activation of the VEGF signaling pathways is the earliest known landmark that defines the endothelial lineage commitment within the nascent mesoderm [20]. CD133 transmembrane glycoprotein was expressed in hematopoietic stem cells and Endothelial Progenitor Cell (EPC). The surface antigen CD133 has been accepted as an alternative EPC marker because it is not expressed on mature endothelial cells. CD133 was rapidly downregulated as progenitors and stem cells differentiate into more mature post-mitotic cells [21]. Interestingly, the staining intensities of CD133 and VEGF were locally strengthened in the inner fields on E6.5-E7.5.Their expression in a spatial distribution suggested patterned networks of channel formation. Therefore, our experiments indicated that embryo cells, upon experiencing a hypoxic environment, increased the expression of HIF-1α at the early stages of embryonic development. As the embryo developed, HIF-1α activated VEGF gene expression. Subsequently, VEGF induced EPC (CD133^+^) embryo cell differentiation as well as the proliferation and tube formation of embryo VM networks on E6.5-E7.5. The expression of VEGF and CD133 were upgraded on E8.5-E9.5. They may promote placenta formation during this time.

Concurrently, HIF-1α may upregulate Twist, which induces a series of biological events [22]. In inner fields, the staining intensity of Twist displays a local plexus on E6.5-E7.5. Twist reactivity in a spatial distribution suggested patterned networks formation, which is consistent with the peak formation of embryo VM on E7.5. The expression of E-cadherin was upregulated and that of Vimentin was downregulated. We also observed that the expression of Twist around the vessels was strengthened. Therefore, we supposed that the Twist relative-protein participated in embryo VM formation, and had a potential role in maintaining embryo cell pluripotency. The expression of Twist enhanced the modality-transmitting ability of embryo mesenchymal cells to endothelium cells. This phenomenon has also been observed in tumor angiogenesis. Twist may promote tumor cell reversion to an embryonic, more plastic phenotype and take part in VM formation of tumors [8, 18].

In summary, HIF-1α plays a deterministic role in the developmental processes as well as tumors. On one hand, HIF-1α promoted VEGF expression, which induces endothelial progenitor cells to differentiate and proliferate. On the other hand, HIF-1α activated the Twist gene, which promotes the transition of mesoderm stem cells in the embryo. In conclusion, HIF-1α promoted the vasculogenesis of the embryo via different ways. The results were consistent with the hypothesis that the genetic ablation of HIF-1α in mice resulted in embryonic lethality at mid-gestation and caused vascularization defects [23].

## Conclusions

Based on our observation and investigation, we described these interesting phenomena and established a model of vessel generation during embryonic development. The LPCA and embryo VM had been formed under local hypoxia environment during the early stage of embryonic growth. LPCA may serve as the spatial foundation for further blood vessel development. Often, understanding of active biological processes during development may suggest molecular mechanisms underlying the neoplastic transformation [24, 25]. This study made provide a solid basis for understanding the biological feature of a tumor. Meanwhile, the study made also provide promising therapeutic target for the treatment of tumor.

## Methods

### Animal and pregnant animal model

Kunming mice (aged 6-8 weeks, 10 males and 25 females) were purchased from the Animal Base of Union Drug Institute (Beijing, China). The average weights were 35-40 g for males and 25-35 g for females. The ratio between females and males in the same cage was 3:1. The noon of the day on which the vaginal plug is found was observed as E0.5 after gestation [26]. The pregnant mice were randomly divided into five groups: E5.5 (5.5 days after pregnancy), E6.5 (6.5 days after pregnancy), E7.5 (7.5 days after pregnancy), E8.5 (8.5 days after pregnancy), and E9.5 (9.5 days after pregnancy). The pregnant mice after completing observation were sacrificed according to the date of pregnancy. All mice were fed in a temperature-controlled room for one week before they copulated. All animals were maintained according to the “Guidelines for the Care and Use of Experimental Animals” established by the Tianjin Medical University, China.

### Immunohistochemistry staining and histochemistry staining

The uterus of pregnant mice were removed, fixed with formalin, embedded in paraffin, cut into 4 μm sections, and mounted onto poly-L-lysine-coated slides. The sections were routinely deparaffinized, and endogenous peroxidase activity was blocked with 3% hydrogen peroxide in 100% methanol for 30 min at room temperature. The sections were rehydrated and washed with Phosphate-Buffered Saline (PBS), and pretreated with citrate buffer (0.01 M citric acid, pH 6.0) for 20 min at 100°C in a microwave oven. After the nonspecific binding sites were blocked using normal goat serum for 30 min at room temperature, the sections were incubated overnight at 4°C with rabbit polyclonal anti-CD31 (1:100 dilution, Abcam, UK), rabbit polyclonal anti-VEGF (1:400 dilution, Thermo Scientific, UK), rabbit polyclonal anti-CD133 (1:100 dilution, Santa Cruz Biotechnology, CA), mouse monoclonal anti-HIF-1α (1:50 dilution, Thermo Scientific, UK), rabbit polyclonal anti-Twist (1:100 dilution, Santa Cruz Biotechnology, CA), rabbit polyclonal anti-E-cadherin (1:100 dilution, Santa Cruz Biotechnology, CA), and rabbit polyclonal anti-Vimentin (1:100 dilution, Epitomics, USA). The sections were then rinsed with PBS, and biotin-labeled secondary antibodies and peroxidase-labeled avidins were applied to the sections at room temperature for 1 hr. The sections were stained with 3,3΄-diaminobenzidine (DAB) chromogen for 5-10 min at room temperature, and washed with distilled water. After IHC staining for CD31, the sections were washed with distilled water for 5 min and incubated with PAS for 15 min. Finally, all sections were counterstained with hematoxylin, dehydrated, and mounted.

Normal human stomach mucous membrane was used as a positive control for PAS staining. PBS was used in place of the primary antibodies as a negative control. The tissue section was used as a positive control according to the antibody instructions. Immunohistochemistry staining results were quantified based on the percentage of positive expression cell area in specific areas.

All sections have been observed by two pathologists and counting performed by them. They did the counting blinded as to the day of gestation.

### Quantification of Microvessel Density (MVD)

According to the protocol introduced by Weidner [7], capillary vessels and microvessels in the embryo stained with CD31 were counted. A single positively stained endothelial cell can be counted as one MVD.

### Quantification of embryo VM

The characteristics of embryonic VM referred to define of tumor VM [6]. The wall of embryo VM was lined with embryonic cells. Red cells can be found in the embryo VM tube. The counting of embryonic VM referred to counting of tumor VM [27]. Embryo VM channels in immunohistochemistry stained sections was counted using × 400 magnification. Five fields were randomly chosen, and the average blood supply pattern was defined as the number of microvessels or channels in one section.

### Quantification of LPCA

The constitution of some embryo cells that were darkly stained in the H & E stained sections were distributed in patterns of lines and networks, and termed as LPCA. The count of LPCA was the percentage of the LPCA cell area in specific areas. LPCA in the H&E staining sections were counted under × 200 magnification. The results presented are mean of the five fields.

### TUNEL staining

For *in situ* visualization of apoptotic cell distribution, we applied the terminal dexynucleotidyl transferase (TdT)-mediated dUTP nick end labeling (TUNEL) staining using an apoptotic cell detection kit (TUN11684817, Roche, USA) following the manufacturer’s directions.

### Statistical analysis

All data in the study were evaluated with SPSS version 13.0 (SPSS Inc., Chicago, IL, USA), and *p* < 0.05 was considered statistically significant. Statistical analysis was performed using one-way ANOVA.

## Abbreviations

Embryo VM: Embryo vasculogenic mimicry
EPC: Endothelial progenitor cell
EPHA2: Erythropoietin-producing hepatocellular carcinoma-A2
HIF-1α: Hypoxia-inducible factor-1α
LPCA: Linearly patterned cell apoptosis
LPPCN: Linearly patterned programmed cell necrosis
MVD: Quantification of microvessel density
PAS: Periodic acid schiff
PBS: Phosphate-buffered saline
TUNEL staining: Terminal dexynucleotidyl transferase (TdT)-mediated dUTP nick end labeling staining
VEGF: Vascular endothelial growth factor
VM: Vasculogenic mimicry.
